# Negative-pressure-related diffuse alveolar hemorrhage after monitored anesthesia care for vertebroplasty: a case report

**DOI:** 10.1186/s13256-021-02697-6

**Published:** 2021-03-16

**Authors:** Yumin Jo, Jagyung Hwang, Jieun Lee, Hansol Kang, Boohwi Hong

**Affiliations:** 1 Department of Anesthesiology and Pain Medicine, Chungnam National University Hospital, 282 Munhwa-ro, Jung-gu, Daejeon, 35015 Korea; 2 Department of Anesthesiology and Pain Medicine, Daejeon Woori Hospital, Daejeon, South Korea

**Keywords:** Ambulatory surgical procedures, Hemoptysis, Interstitial lung diseases, Case report

## Abstract

**Background:**

Diffuse alveolar hemorrhage (DAH) is a rare, life-threatening condition that can present as a spectrum of nonspecific symptoms, ranging from cough, dyspnea, and hemoptysis to severe hypoxemic respiratory failure. Perioperative DAH is frequently caused by negative pressure pulmonary edema resulting from acute airway obstruction, such as laryngospasm, although hemorrhage itself is rare.

**Case presentation:**

This case report describes an unexpected hemoptysis following monitored anesthesia care for vertebroplasty. A 68-year-old Asian woman, with a compression fracture of the third lumbar vertebra was admitted for vertebroplasty. There were no noticeable events during the procedure. After the procedure, the patient was transferred to the postanesthesia care unit (PACU), at which sudden hemoptysis occurred. The suspected airway obstruction may have developed during transfer or immediate arrive in PACU. In postoperative chest x-ray, newly formed perihilar consolidation observed in both lung fields. The patients was transferred to a tertiary medical institution for further evaluation. She diagnosed with DAH for hemoptysis, new pulmonary infiltrates on chest x-ray and anemia. The patient received supportive care and discharged without further events.

**Conclusions:**

Short duration of airway obstruction may cause DAH, it should be considered in the differential diagnosis of postoperative hemoptysis of unknown etiology.

## Introduction

Hemoptysis immediate after non-airway surgery is very disconcerted, and quick identification of cause is important. Diffuse alveolar hemorrhage (DAH) with macroscopic hemoptysis is a rare but life threatening condition characterized by the accumulation within the alveoli of red blood cells arising from the alveolar capillaries. Identifying the cause of DAH is complicated by differences in the clinical presentation of underlying diseases. DAH during the perioperative setting is frequently reported to be caused by negative pressure pulmonary edema resulting from acute airway obstruction.[1, 2] The present study describes a patient who experienced DAH after monitored anesthesia care with sevoflurane while maintaining spontaneous ventilation.

## Case

A 68-year-old Asian woman, 145 cm in height and 45 kg in weight, with a compression fracture of the third lumbar vertebra was admitted for vertebroplasty. Her previous medical history included osteoporosis, and her previous surgical history included appendectomy, removal of a thyroid nodule, and recent repair of the right rotator cuff under general anesthesia. All of the preoperative evaluations were unremarkable except diastolic dysfunction grade I in preoperative echocardiography.

After applying the standard patient monitoring system, including electrocardiography, noninvasive measurement of blood pressure, and pulse oximetry, vertebroplasty was performed with the patient in a prone position. To relieve anxiety, sedation was induced with sevoflurane 8 vol % and oxygen 3L/min via mask ventilation for 2 minutes. Sedation was maintained with sevoflurane of 2~3 vol % with the patient’s head turned sideways. End tidal carbon dioxide (ETCO_2_) was monitored while maintaining spontaneous ventilation.

During vertebroplasty, 6 ml of bone cement (V-STEADY, Sungwon Medical, Korea) was injected through a hollow needle into the fractured bone. There were no noticeable events during the procedure.

**Fig. 1:**
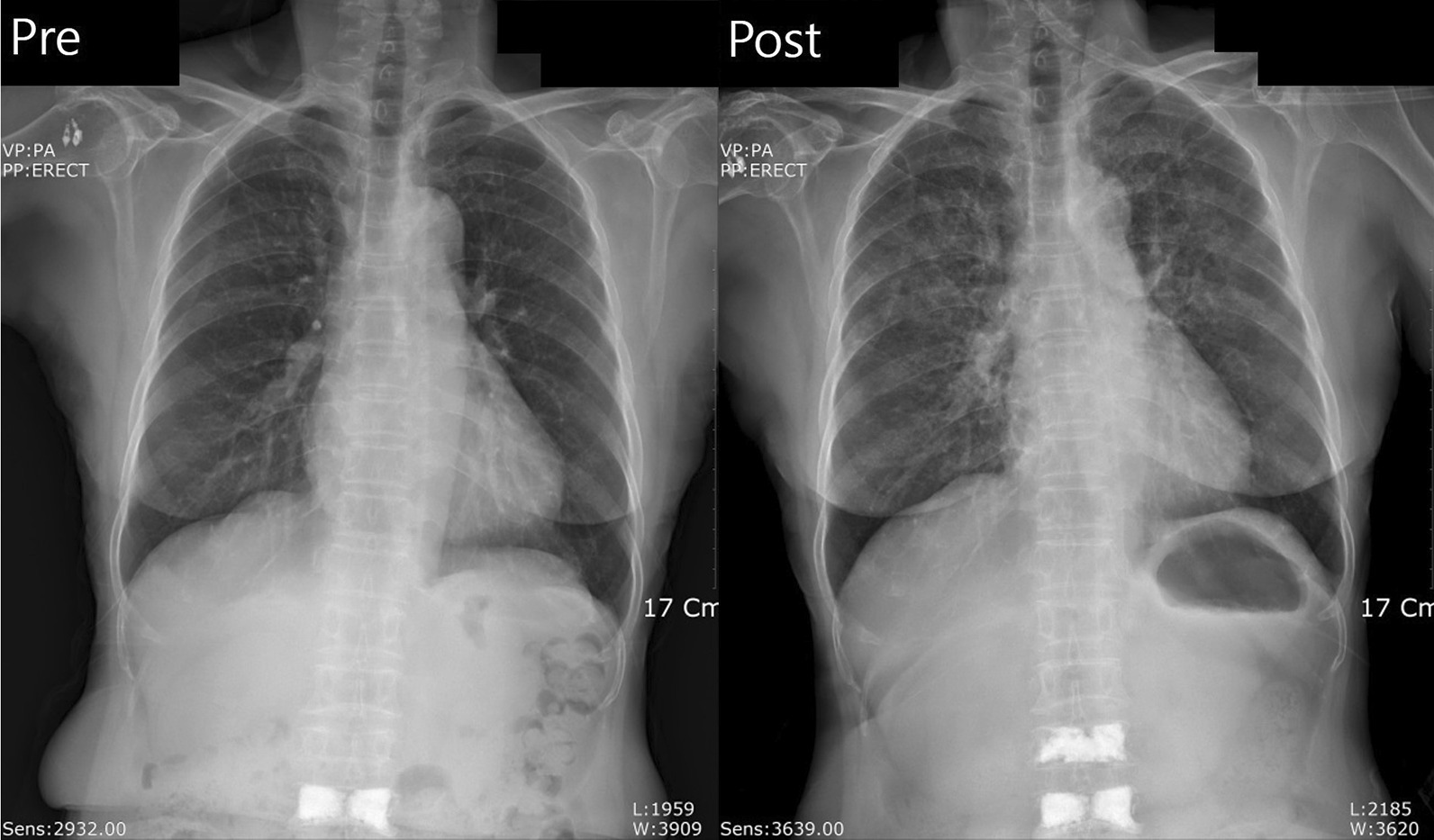
Preoperative and postoperative air bronchograms of the lung fields of the patient. Perihilar consolidation, which was not observed preoperatively, but observed in postoperative images.

After the procedure, the patient was transferred to the postanesthesia care unit, at which sudden hemoptysis occurred. The amount of hemoptysis could not be measured in exact scales, but the amount of frank bleeding was enough to have drenched 4 to 5 sheets of (4 x 4) gauze. As the patient began to show complaints of dyspnea, oxygen saturation was as low as 80 %. After application of 5 liters of oxygen via facial mask, the oxygen saturation showed recovery up to 93%. The vital signs at that time were stable, and no symptoms other than dyspnea complained. Coarse breathing sounds were heard in both lung field. In the postoperative chest x-ray taken immediately afterwards, newly formed perihilar consolidation and air bronchograms which were not present in the preoperative radiograph images were observed in both lung fields (Fig. 1).

**Fig. 2:**
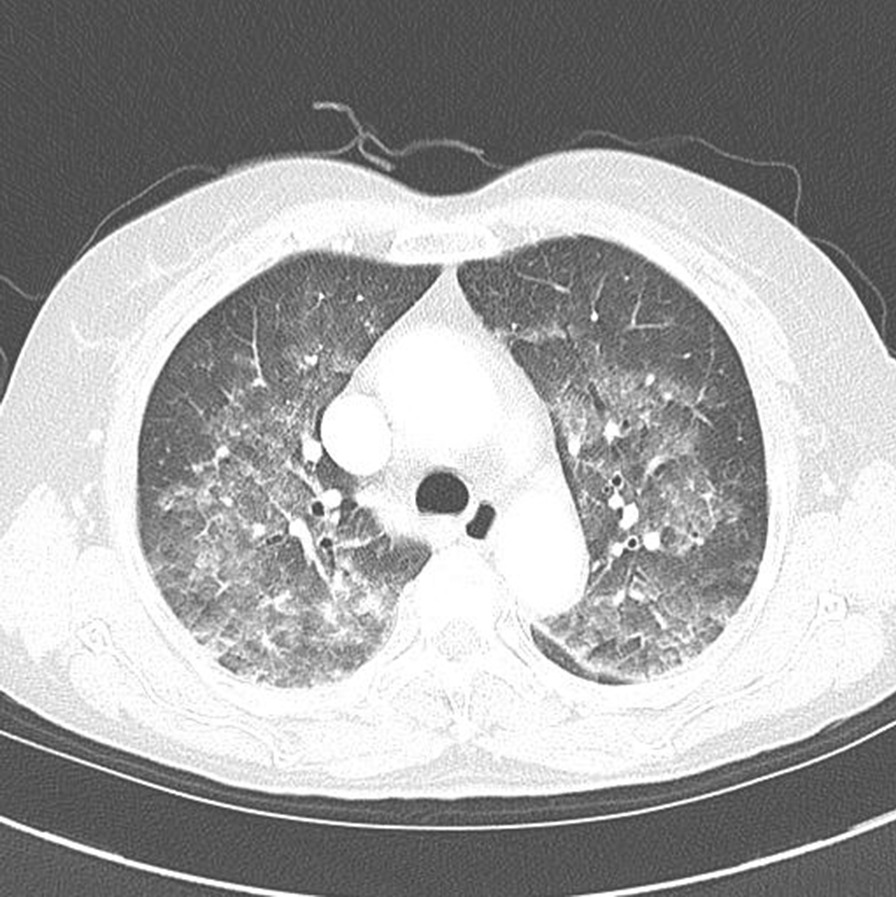
Computed tomography of the central portions of both lungs in the patient, showing diffuse ground glass opacity and interlobular septal thickening.

While the patient insisted that her symptoms were tolerable, she was transferred to a tertiary medical institution for further evaluation. The vital signs at the time of transfer were stable; body temperature of 36.8 °C, pulse rate 82 beats/min, respiration 20/min, and blood pressure 120 / 75 mmHg. The oxygen saturation was 97%. The computed tomography (CT) findings were as stated: diffuse ground glass opacity and interlobular septal thickening in both lungs central portion (Fig. 2). Bronchoscopy was not performed because the symptoms improved and the patient did not want it.

In comparison to the preoperative hemoglobin level of 10.4 g/dL, hemoglobin level decreased to 8.5 mg 2 days after admission. The patient received supportive care and discharged without further events. The patient requested an outpatient clinic follow up, but did not visit until 6 months of discharge.

## Discussion

DAH is a rare but life-threatening condition that can present as a spectrum of nonspecific symptoms, ranging from cough, dyspnea, and hemoptysis to severe hypoxemic respiratory failure requiring mechanical ventilation. [3] DAH results from diffuse damage to the pulmonary small vessels, leading to blood collecting within the alveoli.

The cause of our case can be inferred from many aspects. First of all, we can suspect the cement used in vertebroplasty. Pulmonary cement embolization is a rare but fatal complication of vertebroplasty [4]. If the needle is not properly placed or the injected volume is too large, then cement could result in leak. The multi-focal embolization caused by cement leakage also looks like a DAH. [5] The occurrence of this diffuse lung embolization is only possible when a considerable amount of cement is injected in a very low viscous state. However, radiopaque parts indicating cement could not be found in her CT image, and the leak was not found.

In some cases, DAH occur occasionally in patients with congestive heart failure due to systolic or diastolic left ventricular dysfunction [6]. Increased pulmonary capillary pressure may induce hydrostatic venous pulmonary congestion leading to rupture of the microvasculature with intra-alveolar blood spilling [7]. However, preoperative echocardiography of our patient was non-specific finding except diastolic dysfunction grade I, which means impaired relaxation of left ventricle. That is a near normal finding in individuals by the age of 60.

Although DAH also can be caused by immunological conditions associated with vasculitis [3] and by non-immunological causes, such as infection, malignancy, coagulation disorders, and drug toxicity, [8] perioperative DAH is mainly caused by high negative pressure due to acute airway obstruction. The distinct inspiratory force against an obstructed upper airway triggers high negative intrapleural pressure as well as a large increase in venous return, increasing the cardiac preload. Concomitantly, hypoxia and sympathetic stimulation increase the mean arterial pressure and the afterload, reducing the forward stroke volume. The increased hydrostatic pressure gradient in the pulmonary capillaries leads to transudation of fluid into the alveoli.

A typical perioperative condition is laryngospasm, which occurs after extubation of an endotracheal tube. [9, 10] Other conditions may include biting of the endotracheal tube or the laryngeal mask. [11, 12] Although most patients experience negative pressure pulmonary edema, hemorrhages can develop in the alveoli, and the destruction of pulmonary capillaries plays an important role in overt pulmonary hemorrhage.

Our patient showed no signs of negative pressure pulmonary hemorrhage after spontaneous ventilation, nor evidence suggesting airway obstruction during the vertebroplasty procedure. Continuous intraoperative monitoring of ETCO_2_ confirmed that spontaneous ventilation was well maintained throughout the operation. The suspected airway obstruction may have developed due to the switching of the patient from the prone to the supine position after the completion of surgery and may have occurred temporarily while the patient was transferred into and while she was inside the recovery room.

Alveolar hemorrhage can occur if the intake negative pressure increases rapidly, even during a short period of airway obstruction. A vigorous hiccup may induce a momentary increase in negative pressure of the chest cavity, causing DAH. [13] Even a brief airway blockade by compacted snow can produce a negative pressure pulmonary hemorrhage, as observed in a patient rescued following an avalanche.[14]

Sedation with an inhalational anesthetic is a useful alternative to intravenous administration of sedatives. Sevoflurane alone in air or oxygen can be used to provide conscious sedation. Sevoflurane has a lower blood-gas solubility, which allows for more rapid induction of anesthesia. Nonetheless, high concentrations of sevoflurane are more likely to cause airway obstruction. [15] Additionally, high concentrations of sevoflurane have toxic effects, increasing the risk of alveolar wall damage and, in several cases, inducing diffuse alveolar hemorrhage.[16, 17] However, the mechanism by which sevoflurane causes pulmonary toxicity remains unclear. Lung surfactant damage caused by inhalation toxins can generate severe respiratory problems. Volatile anesthetics are lipophilic compounds that interact with surfactant lipids and promote their dysfunction by biophysical impairment within a short period of time. Lung surfactant, located between the alveolar lumen and the epithelial cell membranes, is the first barrier to any toxic agents that may penetrate into the respiratory system.[18] Sevoflurane may reduce cooperative interactions among phospholipids in the lung surfactant monolayer. Moreover, fluidification of the condensed phases promoted by sevoflurane could potentially impair the ability of surfactant films to sustain the lowest surface tension.[19]

## Conclusion

This case report describes unexpected hemoptysis due to negative pressure pulmonary hemorrhage following monitored anesthesia care with spontaneous ventilation. This uncommon complication should be considered in the differential diagnosis of postoperative hemoptysis of unknown etiology.

## Data Availability

All data generated or analyzed during this study are included in this article.

## Abbreviations

DAH: Diffuse alveolar hemorrhage
ETCO_2_: End tidal carbon dioxide
